# Co-circulating pathogens of humans: a systematic review of mechanistic transmission models

**DOI:** 10.1098/rspb.2025.1453

**Published:** 2025-09-24

**Authors:** Kelsey E. Shaw, Jennifer K. Peterson, Neda Jalali, Saikanth Ratnavale, Manar Alkuzweny, Carly Barbera, Alan Costello, Liam Emerick, Guido Espana, Alex Meyer, Stacy Mowry, Marya Poterek, Carol de Souza Moreira, Eric Lease Morgan, Sean Moore, Alex Perkins

**Affiliations:** ^1^ Department of Biological Sciences, University of Notre Dame, Notre Dame, IN 46556, USA

**Keywords:** co-circulating pathogens, disease transmission, mechanistic modelling

## Abstract

Historically, most mathematical models of infectious disease dynamics have focused on a single pathogen, despite the ubiquity of co-circulating pathogens in the real world. We conducted a systematic review of 326 published papers that included a mechanistic, population-level model of co-circulating human pathogens. We identified the types of pathogens represented in this literature, techniques used and motivations for conducting these studies. We also created an interaction index to quantify the degree to which co-circulating pathogen models include across scale and/or pathogen–pathogen interactions. We found that the emergence of new pathogens, such as HIV and SARS-CoV-2, precipitated modelling activity of the emerging pathogen with established pathogens. Pathogen characteristics also tended to drive modelling activity; for example, HIV suppresses the immune response, eliciting interesting dynamics when it is modelled with other pathogens. The motivations driving these studies were varied but could be divided into two major categories: exploration of dynamics and evaluation of interventions. Future potential avenues of research we identified include investigating the effects of misdiagnosis of clinically similar co-circulating pathogens and characterizing the impacts of one pathogen on public health resources available to curtail the spread of other pathogens.

## 1. Introduction

Mathematical modelling is a powerful tool in understanding and forecasting infectious diseases and shaping public health responses [1–3]. Mathematical models allow researchers to investigate the population-level impact of different treatment regimens for infectious diseases [4], analyse the effect of non-pharmaceutical interventions on disease spread [5] and predict how factors such as climate change will influence transmission of vector-borne diseases [6–8]. However, the majority of infectious disease models focus on a single pathogen, despite the fact that multiple pathogens are co-circulating in any given time and place [9,10].

Co-circulating pathogens have the potential to impact the dynamics of one another via multiple mechanisms. People infected with HIV, for example, are more susceptible to infection by other pathogens, and this individual-level phenomenon can have population-level effects on transmission dynamics [11,12]. HIV, TB and malaria in particular co-circulate widely in under-resourced settings and can exacerbate both the clinical presentation and transmission of one another [13]. This can lead to more interventions necessary at the individual-patient level, as well as a greater population-level burden of disease [14,15]. Furthermore, environmental factors such as climate change and land use changes likely impact multiple pathogens simultaneously and potentially synergistically, which could not be captured with a single-pathogen focus. Deforestation and the expansion of agriculture, for example, have been shown to shift the abundance, diversity and biting rates of mosquito species that are known arbovirus vectors [16,17].

Shifting public health priorities in response to new threats can also have unintended effects on co-circulating pathogens. For example, during the height of the COVID-19 pandemic, routine vaccination campaigns across the world against diseases such as measles and polio were disrupted, with ripple effects on vaccine coverage and transmission dynamics [18–20]. In addition, co-circulating pathogens with similar clinical presentations, such as Zika, dengue and chikungunya, may hinder surveillance efforts to discern the true prevalence of each pathogen contributing to the observed syndromic data due to misdiagnosis and also hampering targeted public health interventions [21–23].

Given the many ways in which co-circulating pathogens may impact each other, mathematical models that explicitly model these interactions may provide key insights. For example, researchers may be better able to capture transmission dynamics and thereby improve forecasting accuracy by including synergistic interactions such as one pathogen increasing host susceptibility to other pathogens [24], or mitigating interactions such as infection causing decreases in host mobility, thus decreasing host exposure to other co-circulating pathogens [25]. The presence of co-circulating pathogens in a population may necessitate different screening or treatment regimens to achieve public health goals, and mathematical models can aide in exploring these different scenarios [26,27]. The myriad possibilities in these approaches motivated us to explore the current state of the literature on mathematical models of co-circulating pathogens.

Despite the evidence for the impact of co-circulating pathogens on each other and the potential usefulness of mathematical models, we did not find any systematic reviews of mathematical models of co-circulating pathogens in our literature search. Therefore, we conducted this systematic review with the goal of characterizing the existing scientific literature on co-circulating pathogen models, to both better understand the current state of this field and identify knowledge gaps that need to be addressed. We narrowed the scope of our review to focus on population-level mechanistic models of human pathogens in order to provide policy-neutral evidence on the state of knowledge in this field that can be used to guide future population-level policy decisions. Specifically, our objectives were:

(i) to determine the most common pathogens modelled together and the ecological, biological and clinical similarities between these pathogens,
(ii) to analyse the authors’ motivations for creating co-circulation models and the intended purpose of their studies,
(iii) to characterize common features of co-circulation models and their outputs,
(iv) to quantify differences between single-pathogen and co-circulating pathogen models, and
(v) to report aspects of co-circulation models that are uncommon in the literature and potentially fruitful avenues for future research.

In undertaking this systematic review, we aim to highlight examples of existing co-circulating pathogen models that provide new advances in understanding of co-circulating pathogen dynamics or guidance for public health policy decisions, as well as identify open questions worthy of future research to fill current knowledge gaps that could better guide future evidence-based policy.

## 2. Methods

### (a) Literature search

**Figure 1.**
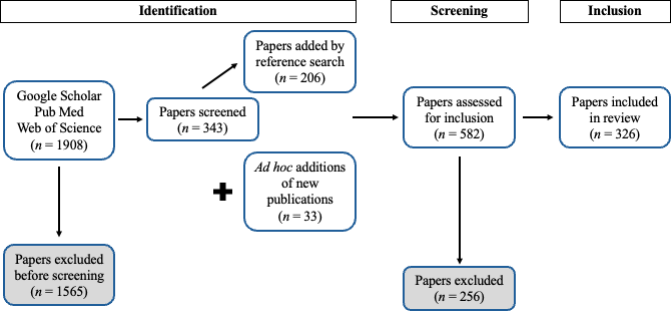
PRISMA (Preferred Reporting Items for Systematic reviews and Meta-Analyses) flow chart for the literature search, screening and inclusion process.

On 21 October 2022 we searched Google Scholar, PubMed and Web of Knowledge databases using a set of keywords. Our keyword list consisted of: co-circulating pathogen; concurrent epidemic; polymicrobial infection AND mathematical model; concurrent infection AND mathematical model; syndemic AND model; co-infection AND mathematical model; heterologous reactivity AND mathematical model; polyparasitism AND mathematical model; synergistic pathogens AND mathematical model; dual infection AND mathematical model; pathogen co-occurrence; co-epidemic; superinfection AND mathematical model. We retrieved a total of 1908 unique bibliographic records from Google Scholar, PubMed and Web of Science, which we reviewed in a four-stage process. In stage one, author J.P. briefly scanned the titles and abstracts of the resulting manuscripts for the inclusion criteria, which yielded a total of 343 papers published between 1985 and 2023 (1565 excluded). In stage two, the references of each selected paper were then scanned by author J.P. to find additional publications, which yielded an additional 206 papers. Additionally, any newly published and relevant papers that the authors became aware of during the review process were added (33 papers total). In stage three, a pair of reviewers assessed each paper (*n* = 582) for meeting our inclusion criteria (below) and reached a consensus. In stage four, the reviewers first extracted the data separately and then later compared their questionnaire answers to reach a consensus on each question. We excluded papers not published in English (figure 1).

### (b) Inclusion criteria

All 582 papers were evaluated for inclusion based on six questions:

(i) is the paper an original research article?
(ii) are at least two real pathogen species (not theoretical) modelled in the same model?
(iii) does at least one of the pathogens modelled cause human disease?
(iv) does the model have at least one population-level aspect in a human population?
(v) is some part of the model structure informed by a mechanistic derivation of biological processes as opposed to purely involving phenomenological relationships?
(vi)is there a place in the model where hosts transition from a susceptible to an exposed or infected state?

We narrowed the scope of this review to real pathogen species that cause human disease (criteria ii and iii) because co-circulating pathogens have been a salient topic in the non-human literature for a longer period, and we were interested in how that has percolated into the modelling of human disease. We focused on mechanistic, population-level models that include a transmission process (criteria iv–vi) in contrast to descriptive or statistical models that fit data without any consideration of the biological mechanisms involved. We defined a mechanistic model (criteria v) as one that explicitly includes relevant biological processes, such as host susceptibility or parasite virulence, in the model construction while phenomenological models may examine outcomes such as disease prevalence but only via statistical methods that do not include a biological process. While non-mechanistic models can provide meaningful information for population-level policy decisions, we were particularly interested in exploring the state of the mechanistic literature because many policy decisions may rely on interrogating the underlying biological processes dictating the interactions between multiple pathogens. Furthermore, by restricting our analysis to this group of models, we were able to make comparisons within our dataset that would not be meaningful across mechanistic and non-mechanistic models. We included papers for which the answer to all six questions was ‘yes’.

### (c) Questionnaire development

We used a questionnaire to screen each publication in order to standardize the data generated from each review. We developed the questionnaire through a collaborative, iterative three-stage process. In the first stage of the development process, our aim was to evaluate the ability of the questionnaire to elicit similar responses from multiple reviewers assessing the same paper. To this end, reviewers used the questionnaire to review the same paper, and we then evaluated response data for discrepancies. Through group discussion, we identified areas of improvement, and feedback from the group was applied to the next iteration of the questionnaire. We carried out this process twice. Finally, in the paper review process, all papers were reviewed in pairs as described below and the data generated were used for analysis. All reviewers were in communication throughout the review stage to harmonize any discrepancies that arose.

### (d) Paper review process

Two reviewers were randomly assigned to each publication, which they reviewed using the standardized questionnaire. After reviewing each paper individually, the pair of reviewers assigned to each paper then met to compare and discuss answers and resolve any discrepancies between answers. Each pair then submitted a final version of their answers in the form of a third survey marked ‘consensus’. Only consensus surveys were analysed (*n* = 326).

### (e) Questionnaire description

The questionnaire consisted of six sections: (i) bibliographic information, (ii) inclusion criteria, (iii) basic information about each parasite modelled, (iv) model details, (v) model outputs, and (vi) motivation for selecting the pathogens modelled. The final version of the questionnaire contained 47 questions.

### (f) Author motivations

Question 39 of our systematic survey was an open-response question: ‘Did the authors develop their model to answer a specific research question or to achieve a specific purpose or objective?’ To analyse the responses to this question, we inserted the pooled responses to this question into ChatGPT v.3.5 [28] with the prompt ‘The text I provided you was supposed to illustrate authors’ motivations to write the papers we analysed. From that text, what would you say are the main motivations of authors?’ Authors K.S. and T.A.P. then discussed the output from ChatGPT and refined the language of the motivations ChatGPT produced to create four categories of motivations, and K.S. categorized each response to question 39 according to these motivations. Each response could receive more than one categorization.

### (g) Bibliometrics

To explore how journal prestige and topic may be related to citation patterns, for all papers included in our review for which a DOI was available, we queried OpenAlex [29] to collect the times each paper has been cited as well as the h-index and i−10index for the journal in which each paper was published, as of 11 March 2024. We then used the glmmTMB package in R [30] to create generalized linear models to assess potential associations between citation count and publication year, journal h-index and journal i−10index. We also used the Web of Science API Lite [31] to collect subject categories for each paper. We then binned related categories such as ‘Mathematics, Applied’ and ‘Mathematics’ to create seven meta-categories. We created a second generalized linear model to test for the impact of assigned meta-category on citation counts.

### (h) Interaction index

To estimate the extent to which each co-circulating pathogen model in our dataset accounted for interactions either across scale, e.g. from within-host to population level, and/or interactions between pathogens such as one pathogen changing host susceptibility to another (electronic supplementary material, Box S1), we designed an interaction index derived from the responses to questions 23−30 in the questionnaire. The index was calculated by assigning one point to each answer that indicated an interaction and then totalling the points. For example, question 23 (‘Does the model structure include hosts that are co-infected with two or more pathogens?’) would have yielded one point for a ‘yes’ answer and zero points for a ‘no’. For multi-part questions such as question 23 f (‘Which parameter values for the co-infected class are different from the mono-infected and uninfected classes? (select all that apply)’), we assigned one point for each parameter value selected. The maximum possible interaction index score was 18. An example of how this index was calculated can be found in electronic supplementary material, table S1.

### (i) Data availability

A full version of the questionnaire can be found in the supplementary materials. All data were analysed in R v. 4.3.0 [32], and raw files and code used for analysis as well as a supplementary file of all tables produced from the analysis are available at https://figshare.com/s/2b268f5df8aed6acad3f.

## 3. Results

### (a) Study selection

**Figure 2.**
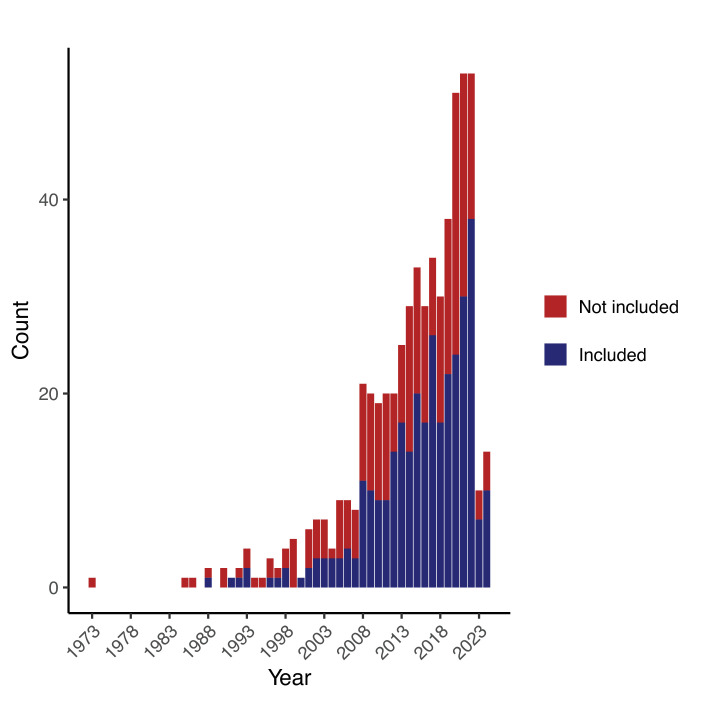
Temporal trends in the publication of co-circulating pathogen models. Papers that met our inclusion criteria gradually increased over time. The year of 2024 was only partially sampled up to papers published by the month of October.

In our four-stage review process, we identified 582 papers to review for inclusion, 326 of which met our inclusion criteria (figure 1). We analysed the exclusion cause(s) of the 256 papers which made it to the ‘screening’ step (figure 1) but then did not meet inclusion criteria. The most common reasons for paper exclusion were: (i) the study did not include two real pathogen species in the same model (70.4%, 171/256, question 7), (ii) there was no place in the model where hosts transitioned into an exposed or infected state (45.7%, 111/256, question 11), and (iii) the model did not have at least one population-level aspect (37.4%, 91/256, question 9). Some papers were excluded based on multiple criteria. The oldest papers that met our search criteria were from 1988, although the majority of relevant papers were published in 2008 or later (90.5%; 295/326, figure 2).

### (b) Pathogen information

**Figure 3.**
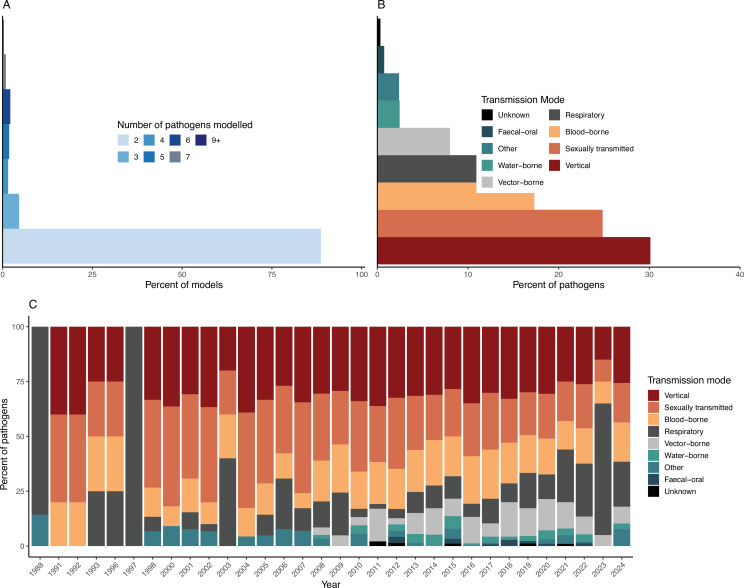
Pathogen characteristics. (A) The frequency of how many pathogens were included in models. The vast majority of models included two pathogens. (B) Frequency of transmission modes represented across all pathogens modelled. Vertically transmitted pathogens, blood-borne pathogens and sexually transmitted pathogens were common in our dataset due to the large number of models focused on HIV. (C) Change in transmission modes represented in our dataset over time, represented as a percentage of the total models we analysed for that publication year.

The majority of included models considered two pathogens (88.6%, 289/326), while the maximum number of pathogens included in the same model was nine (0.31%, 1/326; figure 3A). The most commonly modelled pathogen pairs were human immunodeficiency virus (HIV) and *Mycobacterium tuberculosis* (TB), representing 25.5% (83/326) of the models we reviewed. Other models that included more than two pathogens often were focused either on STIs (e.g. [33–35]) or vector-borne diseases such as dengue, chikungunya and Zika that are transmitted by the same vector (e.g. [36,37]). The one model that included nine pathogens considered a panoply of common STIs: HIV, genital herpes, syphilis, cancroid, gonorrhoea, chlamydia, trichomonas, bacterial vaginosis and vaginal candidiasis [38].

To investigate which modes of transmission were included in the same models, we recorded all viable transmission modes for each pathogen; e.g. HIV would be recorded as ‘sexually transmitted’, ‘vertical’ and ‘blood-borne’. Pathogens were assigned modes of transmission based on all known modes for each pathogen, irrespective of whether all modes were explicitly included in the model structure. We chose to include all known modes of transmission for three reasons: (i) for this analysis we were focused on the characteristics of the pathogens in our dataset, not specifically in how they were modelled, (ii) for pathogens with multiple possible transmission modes, many models used a generic contact process in their model construction without specifying the transmission mode, and (iii) for many of the models, even if only one mode was included in the model structure, some of the papers’ conclusions could be relevant to future work on other known transmission modes for the same pathogens. Therefore, when we calculated the percentage of each transmission mode in our data, the denominator was the total frequency of all modes of transmission (1461). As a result, the percentage calculated does not correspond to the transmission modes found in the models in our dataset, but rather the per cent of biologically known modes attributed to the modelled pathogens. Corresponding to the exceptionally high number of HIV-TB models, the most common transmission modes of pathogens were vertical (30.1%, 440/1461), sexually transmitted (24.8%, 363/1461) and blood-borne (17.3%, 253/1461; figure 3B). Respiratory transmission was also common at 13.8% (201/1461). We also found that the earliest papers in our dataset tended to focus on sexually transmitted and respiratory pathogens, again reflecting the focus on HIV in combination with other STIs and with TB in the 1990s (figure 3C). Over time, a greater diversity of transmission modes appeared, notably with an increase in models of vector-borne diseases starting in 2008 and an increase in models with respiratory pathogens starting in 2020, coinciding with the SARS-CoV-2 pandemic.

**Figure 4.**
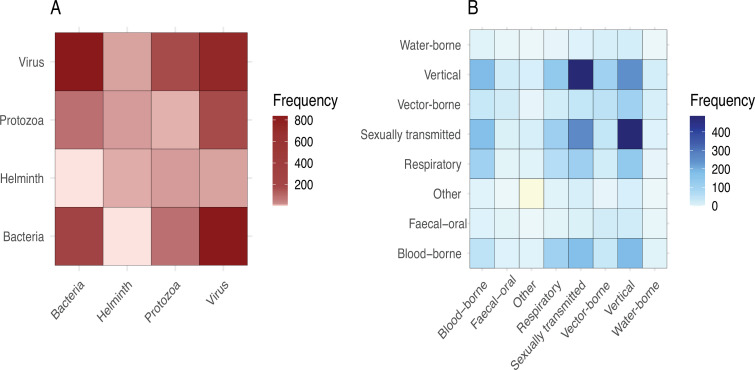
Frequency of modelling pathogens within and between taxonomic groups (A) and transmission modes (B).(A) Viruses and bacteria were modelled together most frequently. (B) Sexually transmitted pathogens were modelled together most frequently with vertically transmitted pathogens and with respiratory pathogens. Light yellow indicates zero models that paired two pathogens with a transmission mode of ‘Other’.

Beyond individual pathogens, we also investigated the frequency with which different pathogen taxa and different transmission modes were modelled together. We categorized the taxonomic group and transmission mode for each pair of pathogens in our dataset and counted the frequency of their co-occurrence in the same model (figure 4). We categorized taxonomic groups as either virus, bacteria, helminth, protozoa or fungus. We categorized transmission modes as vector-borne, sexually transmitted, respiratory, faecal-oral, water-borne, blood-borne, vertical or ‘other’. Examples of transmission modes that fell into the ‘other’ category are direct contact with lesions and soil-transmitted pathogens. This analysis reflected all possible transmission routes for each pathogen and also all possible pairings within the same model. As such, a single paper could be represented multiple times based on the number of pathogens and transmission modes present, resulting in 156 unique pathogen–pathogen pairs and 607 pathogen pairs total. Once categorized, there were 22 distinct taxonomic pairs (607 total) and 69 distinct transmission mode pairs (2206 total). Viruses and bacteria (26.2%, 159/607) and viruses with other viruses (24.5%, 149/607) occurred together most commonly in models. Vertically transmitted and sexually transmitted pathogens (12.2%, 270/2206) and sexually transmitted pathogens with other sexually transmitted pathogens (11.9%, 263/2206) were modelled together most frequently, likely reflecting the large number of STI-focused models. Conversely, some pathogen pairs were extremely rare in our dataset: for example, bacteria and helminths were only modelled together in 0.5% (3/607) of taxonomic pairs, and water-borne pathogens with other water-borne pathogens (0.045%, 1/2206) and faecal-oral pathogens with water-borne pathogens (0.091%, 2/2,206) were rare as well.

To assess which pathogens modelled together shared ecological similarities, we defined this term as ‘pertaining to anything that deals with the relations of organisms (including pathogens) to one another or to their physical surroundings’. We found that the majority of pathogens modelled together did not share ecological similarities, as reported by authors (85.9%, 280/326, question 40). Of those that did, 56.5% (26/46) had shared seasonality and 32.6% (15/46) had shared environmental drivers of transmission. Shared seasonality was commonly noted for respiratory pathogens such as SARS-CoV-2, influenza and RSV. Shared environmental drivers were noted for pathogens such as malaria and cholera, which are both sensitive to environmental conditions (e.g. rainfall) but via different mechanisms [39,40]. Furthermore, pathogens that shared both seasonality and environmental drivers were noted for several models (9/46, 19.6%, electronic supplementary material, figure S1A). Multiple ecological similarities were most often recorded for models of vector-borne pathogens with a shared vector taxa, such as the dengue, chikungunya and Zika viruses.

In contrast, a majority of pathogens modelled together did share at least one biological similarity (56.1%, 183/326, question 41), with 67.2% sharing a transmission route (123/183) and 60.7% (111/183) sharing host subsets as the most common responses. Shared transmission routes were common in models with sexually transmitted pathogens, vector-borne pathogens and respiratory pathogens. Models with shared host subsets commonly included studies of intravenous drug users [41–43] and childhood diseases [25,44]. Clinical or epidemiological similarities were shared by 63.5% (207/326, question 42) of pathogens modelled together, with the most common similarity being that treatments existed for both pathogens (58.9%, 122/207), due to the large number of HIV-TB and STI models for treatable pathogens.

### (c) Motivations and purpose

Reviewers identified two main scholarly motivators (question 45): (i) prior research suggested an effect of one pathogen on another but more research is needed (55.2%, 180/326), and (ii) few or no prior modelling studies conducted of these two pathogens together (35%, 114/326). No clear scholarly motivation was noted in 11% (36/326) of papers.

In addition, we used ChatGPT (v.3.5) (‘ChatGPT’ 2024) to summarize common themes in the reviewers’ answers to the open-ended question 39, ‘Did the authors develop their model to answer a specific research question or to achieve a specific purpose or objective?’ (Box 1). We then refined the results from ChatGPT to better reflect the nuances we felt existed in our dataset, resulting in four categories of author motivation. We then classified reviewer responses to question 39 according to the four motivation categories, allowing for more than one category to apply to each paper. The categories that resulted from this process were:

(i) Understanding the dynamics of co-circulating pathogens, in particular, how multiple pathogens may alter the transmission patterns and/or population outcomes of one another. This category applied to papers in which the authors’ goals included evaluating quantitative outcomes such as *R*_0_, disease burden, mortality, etc., and how outcomes differed in the presence of co-circulating pathogens.
(ii) Developing mathematical models for specific pairs of pathogens of interest, often due to the perceived novelty of doing so by the authors. While all papers in this review included a mathematical model, this category was for those that had a primarily mathematical focus or in which the authors only stated motivation for developing the model was that the pathogens had not been previously modelled together.
(iii) Implementing interventions or control measures for one pathogen and the resulting impact on it and/or other pathogens in the model. We placed papers in this category when the authors were investigating how interventions such as treatment, vaccination or quarantining would impact co-circulating pathogens through either direct or indirect effects.
(iv) Calculating cost-effectiveness and optimal control of different treatment and control strategies. While there was some overlap with (iii), we created this category to capture papers in which the authors were not necessarily investigating the impact of interventions on outcomes such as transmission or mortality, but in which they were primarily concerned with the best way to implement interventions given cost or labour constraints.

Box 1. **Large language learning model analysis of survey question 39, ‘Did the authors develop their model to answer a specific research question or to achieve a specific purpose or objective?’**

We provided the reviewers’ answers to question 39 for all papers included in our review that met inclusion criteria and provided the prompt ‘The text I provided you was supposed to illustrate authors’ motivations to write the papers we analysed. From that text, what would you say are the main motivations of authors?’ The exact output created, prior to our modified version stated in the text, was as follows:

(1) ‘Understanding disease dynamics: authors aim to gain a deeper understanding of the dynamics of infectious diseases, including their transmission patterns, interactions between different pathogens and the impact of interventions on disease spread.
(2) Developing mathematical models: many authors are motivated to develop mathematical models to simulate disease transmission, assess the effectiveness of intervention strategies and predict the outcomes of various scenarios. These models allow researchers to study complex disease dynamics and inform public health decision-making.
(3) Evaluating intervention strategies: there is a strong emphasis on evaluating the effectiveness and cost-effectiveness of intervention strategies, such as vaccination programmes, treatment protocols and preventive measures. Authors seek to identify optimal strategies for controlling disease spread and reducing disease burden in populations’.

Category (i), understanding disease dynamics, was the most common motivation for creating the models, with 51.8% (169/326) of papers falling into this category. Implementation of interventions (iii) was the second most common, at 35% (114/326), followed by cost-effectiveness (iv, 15.6%, 51/326) and developing mathematical models (ii, 15.6%, 51/326). Overlapping categorizations were common, in particular for categories (i) and (iii) (electronic supplementary material S2). Some papers fell into one or more of the four categories but also contained unique motivations and thus were counted in one or more of the four main categories and additionally as ‘other’ (9.5%, 31/326). A small number, 1.5% (5/326), did not neatly fit into any of these categories and were categorized only as ‘other’. Examples of ‘other’ author motivations include examining how one pathogen may exert indirect evolutionary pressures on another [45], or what fundamentally allows for the co-existence of two co-circulating pathogens [46].

### (d) Bibliometrics

**Figure 5.**
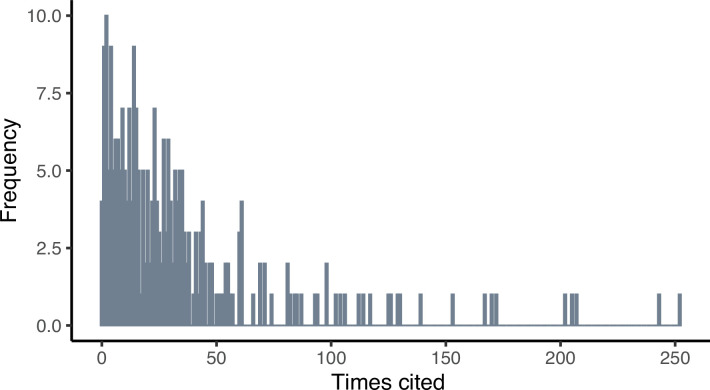
Frequency of citations. Most papers in our dataset were cited fewer than 30 times, however there were several outliers with over 100 citations.

We were able to source bibliometric data for 261 out of 326 of the papers in our analysis. The majority of these papers were cited fewer than 30 times (60.15%, 157/261, figure 5). However, there were some distinct outliers, with the most highly cited paper having 252 citations (table 1). Year of publication (*z* value = −8.93, *p* < 0.001) and h-index (*z* value = 9.32, *p* < 0.001) of the journal in which a paper was published were both significantly associated with citations, but the effect sizes were minimal (central estimates of −0.0564 per year and 0.00243 per h-index unit, respectively), suggesting that outliers in citations likely reflect papers that were particularly useful or interesting to the field.

**Table 1.** Top 10 cited papers from our dataset.

Title	Journal	DOI	Times cited
Genital herpes has played a more important role than any other sexually transmitted infection in driving HIV prevalence in Africa	*PLOS One*	10.1371/journal.pone.0002230	252
Modelling the impact of global tuberculosis control strategies	*Proceedings of the National Academy of Sciences*	10.1073/pnas.95.23.13881	243
Incidence of gonorrhoea and chlamydia following human immunodeficiency virus pre-exposure prophylaxis among men who have sex with men: a modelling study	*Clinical Infectious Diseases*	10.1093/cid/cix439	207
Mathematical analysis of the transmission dynamics of HIV/TB coinfection in the presence of treatment	*Mathematical Biosciences and Engineering*	10.3934/mbe.2008.5.145	205
Oscillations and chaos in epidemics: a nonlinear dynamic study of six childhood diseases in Copenhagen, Denmark	*Theoretical Population Biology*	10.1016/0040−5809(88)90019−6	202
Mathematical analysis of a model for HIV-malaria co-infection	*Mathematical Biosciences & Engineering*	10.3934/mbe.2009.6.333	172
Comparing dengue and chikungunya emergence and endemic transmission in *A. aegypti* and *A. albopictus*	*Journal of Theoretical Biology*	10.1016 /j.jtbi.2014.04.033	170
Ecological interference between fatal diseases	*Nature*	10.1038/nature01542	167
Determinants of the impact of sexually transmitted infection treatment on prevention of HIV infection: a synthesis of evidence from the Mwanza, Rakai, and Masaka Intervention Trials	*The Journal of Infectious Diseases*	10.1086/425274	153
Identifying the interaction between influenza and pneumococcal pneumonia using incidence data	*Science Translational Medicine*	10.1126/scitranslmed.3005982	126

We found additional metadata for 296 of our 326 papers on the Web of Science search engine. This metadata provided subject categories for each paper, with one to five categorizations per paper. By binning related categories (for example, ‘Mathematics, Applied’ and ‘Mathematics’; electronic supplementary material, table S2), we created seven main categories related to the research subject areas for each paper. Life sciences (51.7%, 153/296) and Mathematics (49.7%, 147/296) were the most common categories, while Operations and Management was the least common (2.7%, 8/296). Overlap between categories, in particular Mathematics and Life Sciences, was also common (electronic supplementary material, figure S3). Category was not significantly associated with citations.

### (e) Model characteristics and outputs

The majority of publications presented deterministic models (89.9%, 293/326), followed by individual-based models (8.0%, 26/326). A very small number of papers contained a within-host model in addition to the population-level model required to meet our inclusion criteria (3.7%, 12/326). Spatially explicit models and metapopulation models were rare among the papers reviewed (0.61%, 2/326 and 0.31%, 1/326, respectively). Some papers contained multiple model components with varied structures, and thus fell into multiple categories (9.8%, 32/326).

A total of 85.3% of the publications included models that were informed by data (278/326). Of these papers, 32.0% (89/278) used data extracted from the literature without giving further information on the origin of those data. Surveillance data were used in 28.8% (80/278) of papers, 9.7% (27/278) used survey data and 2.5% (7/278) used case study data. The most common use of data was for model parameterization (96.4%, 268/278), defined as the use of data-informed parameter values taken from other modelling studies. A smaller set of papers also used data for model fitting or calibration (49.2%, 137/278).

The most commonly reported model output (question 35) was disease prevalence (81%, 264/326), followed by the reproduction number (61.7%, 201/326) and stability properties of the model (60.4%, 197/326). Several model outputs were not initially captured by our survey options and were coded as ‘other’ (50%, 163/326). Among these, common responses we found in the ‘other’ category were model sensitivity analysis, cost evaluation of different interventions and total cases of disease averted by interventions. Outputs were commonly examined as a function of different interventions (question 36, 47.2%, 154/326), varying parameter values (22.7%, 74/326) and time (22.4%, 73/326). Rarely did models evaluate outputs as a function of seasonality (0.92%, 3/326), space (0.31%, 1/326) or temperature/climate (0.31%, 1/326).

### (f) Interaction index

**Figure 6.**
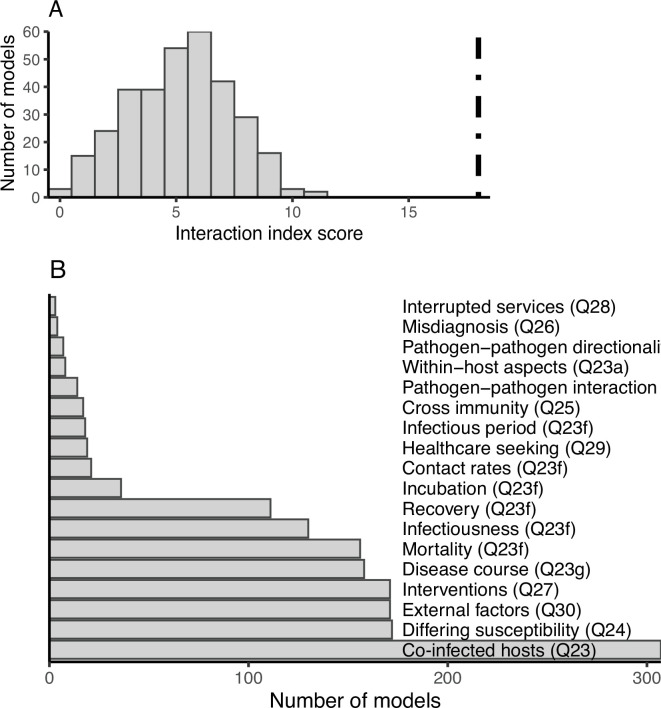
Measures of model interaction. We calculated an interaction index, in which a model received one point for each interaction either across scale and/or between pathogens. (A) The frequency of interaction scores in our dataset; black line represents the maximum possible score of 18. Mid-range scores between 3 and 7 were most common. (B) The frequency of each interaction component in our dataset.

We created an interaction index to quantify how much co-circulating pathogen models accounted for interactions across scale and/or between pathogens (electronic supplementary material, Box S1). Scores were assigned based on questions 23−30, with one point for each interaction (see §2.h). The largest score on the interaction index we observed was 11, out of a maximum possible value of 18. Scores in the intermediate range of 3−7 were most common (71.8%, 234/326), with very few at the minimum score of 0 (3/326) or the maximum observed value of 11 (2/326; figure 6A). This middle range of scores reflected a pattern we commonly observed where most models aimed to investigate one or two dimensions of interaction between pathogens, but upon the incorporation of a second (or more) pathogen(s), the known biology of the interplay between the pathogens often required the inclusion of multiple interactions. For example, models of pathogens that co-circulate with HIV included the increased susceptibility of HIV-infected people to other pathogens even if that was not the focus of the model. The most commonly observed interactions were including hosts that were co-infected (98.7%, 307/326, question 23), including external factors that affect the transmission of both pathogens (e.g. interventions) (54.9%, 171/326, question 30), and allowing mono-infected hosts to have different levels of susceptibility to other pathogen(s) in the model as compared with uninfected hosts (55.3%, 172/326, question 24). The least commonly observed interactions were accounting for interruptions or declines in services offered due to another co-circulating pathogen in the model structure (0.96%, 3/326, question 28) and the model structure including reporting or surveillance errors due to misdiagnosis between the pathogens modelled (1.3%, 4/326, question 26). Models that included across scale interactions such as within-host aspects of co-infection in the model structure were also rare (2.6%, 8/326, question 23a; figure 6B).

**Figure 7.**
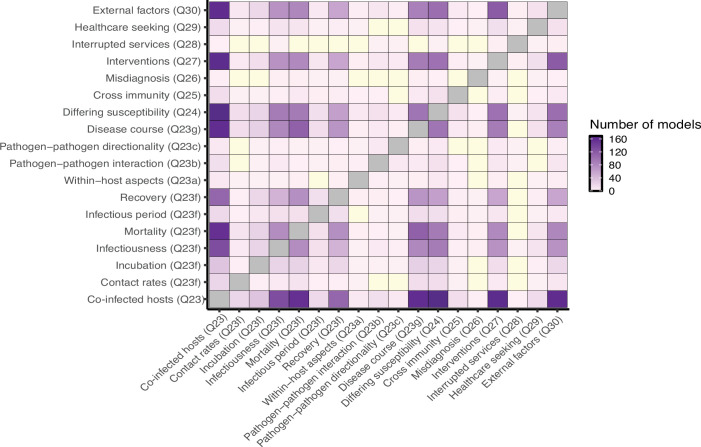
The co-occurrence of different interactions within the same model. The inclusion of co-infected hosts and external interventions, in particular interventions, was very common in our dataset. Combinations that did not occur in our dataset are in yellow.

Certain components of our interaction index were more likely to be included in the same model than others. Including co-infected hosts (question 23) was the most common component in our dataset, and thus also resulted in the most interactions with other components. Including co-infected hosts (question 23) and differing levels of susceptibility to other pathogens between uninfected and mono-infected hosts (question 24) were observed together more than any other pair of interactions (54.3%, 169/326), as well as incorporating both co-infected hosts (question 23) and interventions (question 27) (52.1%, 162/326) and incorporating co-infected hosts and external factors impacting transmission (question 30) (51.1%, 159/326) (figure 7A). When looking at the correlation of different interactions (either jointly present or absent in a model), the most highly correlated pairs were likely due to question redundancy and dependency in our survey. For example, questions 23a, ‘Does the model structure explicitly include within-host pathogen–pathogen interaction?’ and 23b ‘What type of pathogen–pathogen interaction is represented?’ are highly correlated (*r* = 0.7). Other than these types of related questions, there were few very strong correlations, either positive or negative, indicating high variation in which aspects of pathogen–pathogen interactions models in our dataset elaborated on (figure 7B).

## 4. Discussion

Pathogens do not exist in a vacuum; they are part of a complex ecosystem that, among other players, contains multiple pathogens that often vie for the same pool of susceptible hosts and can be influenced by the same external factors, such as climate and vector abundance. Mathematical models that explicitly explore the dynamics of co-circulating pathogens have the potential to inform numerous open questions in disease ecology and public health policy. In our systematic review of co-circulating pathogen models, we found that the scope of the literature in this field has gradually expanded over the past three decades. The emergence of HIV and the clear connection between HIV infection and increased susceptibility to other pathogens was a major catalyzing event for this area of work. However, even as models of multiple pathogens have become more common, there are still extensions of these models that remain underexplored, such as tying within-host processes to population-level models, exploring the effects of misdiagnosis of clinically similar diseases and assessing the impacts of changes in resource allocation during outbreaks.

### (a) Modelling interactions in co-circulating pathogens

We created an interaction index to capture the extent to which a co-circulating pathogen model included interactions either across biological scales and/or between pathogens, and found that the majority of models in our review fell into a middle range of this index. The inclusion of a second pathogen in the model often necessitated a few key additional parameters to capture the known biological interactions between the two pathogens, but models rarely deployed the full range of potential pathogen–pathogen interactions in their construction. For example, including HIV and TB in the same model necessitates that the model structure account for increased susceptibility to TB in HIV-infected hosts. Other interactions between the two pathogens also commonly included a higher likelihood of TB reactivation from the latent phase in HIV-infected individuals and increased morbidity and mortality in HIV-TB co-infecteds. Far less common in our dataset was the inclusion of external factors that reflect the ways in which our health systems interface with the world of co-circulating pathogens and their hosts. For example, arboviruses such as dengue, chikungunya and Zika can present with similar clinical symptoms, which can lead to misdiagnosis of one infection for one another [47]. Misdiagnoses can then result in spurious surveillance data from which we build our models [21], and potentially mis-appropriated public health funds targeted to prevent one disease at the expense of the others. Future work investigating these clinical misdiagnoses could better guide policies governing surveillance and government intervention campaigns for arboviruses. Another topic that came hurtling to the foreground with the COVID-19 pandemic is the potential of one emerging pathogen to interrupt and delay healthcare services targeted towards the treatment and prevention of other pathogens [20]. During the COVID-19 response, routine vaccination and surveillance programmes for endemic diseases were put on hold in many nations, with negative consequences, especially for children [19,48].

### (b) A new framework for model development

Our analysis of author’s motivations resulted in binning papers into four meta-categories: (i) understanding the dynamics of co-circulating pathogens, (ii) developing novel mathematical models, (iii) implementing intervention or control measures, and (iv) calculating cost-effectiveness and optimal control of interventions. As an overarching schema to approach the current state of the field, we propose that papers in categories (i) and (ii) be classified under an umbrella of **exploration** and those in (iii) and (iv) as **evaluation**. This overarching classification provides a convenient framework to be used for both viewing the current state of the literature and evaluating where knowledge gaps remain. Many of the papers focused on exploration had a primarily mathematical goal to simply develop a compartmental model of two co-circulating pathogens [49,50], but some extended into testing more fundamental hypotheses. For example, a model exploring why yellow fever and dengue can co-exist in some regions and not others supported two complementary hypotheses: that cross-immunity between yellow fever and other flaviviruses, including dengue, decreases the pool of susceptible hosts and that competition between vector species allows for continued co-circulation [46]. Another example of a model focused on exploration demonstrated how asymmetric cross-immunity between related *Bordatella* species observed in laboratory studies could result in dominance of *B. pertussis* due to a competitive advantage, and that *B. pertussis* vaccination campaigns would likely not lead to a decrease in *B. parapertussis* burden [51]. One notable example of evaluation included exploring the trade-off between decreased population-level transmission of tuberculosis with extended drug therapy regimens versus the selective pressure for drug-resistant strains, drawing the conclusion that extending access to a specific drug, bedaquiline, to more patients with multi-drug-resistant TB may result in increased resistance to bedaquiline but would still have an overall positive impact on secondary cases and life years lost [52]. Another demonstrated that a lack of interventions due to health services disruptions from the COVID-19 pandemic could have devastating impacts on HIV, malaria and tuberculosis morbidity and mortality over the following 5 years [53]. In general, models with a focus on evaluation are particularly well-suited to provide the needed evidence base for those formulating and assessing public health policies such as designing mass drug administration campaigns or vaccination recommendations. Overall, this framework of exploration and evaluation can be useful for researchers to take stock of the current state of knowledge in their system and identify critical knowledge gaps to push the field forward.

### (c) Model innovation and scope

Despite the number of yet unexplored avenues in the field of co-circulating pathogens left to pursue, in our review, we noted that there was a glut of papers with very similar features that perhaps represent a saturation of this corner of the field. Specifically, 39.9% (130/326) of papers included two pathogens and had near-identical results sections that calculated the disease-free equilibria in the model, the basic reproduction number and performed one or two simple numerical simulations of host prevalence. These same models often fully lacked any data to inform the model (26.9%, 35/130), or used recycled parameters from the literature (74.6%, 97/130). Although there is value in such mathematical approaches, each additional study that performs these steps on a nearly identical system makes only a very incremental contribution to the body of knowledge of co-circulating pathogen dynamics. Furthermore, we found that certain types of pathogens, such as sexually transmitted pathogens were much more highly represented in our dataset while there were many potential pathogen combinations that remain relatively unexplored in the literature.

Another issue we frequently noted was a mismatch between the known pathogen–pathogen interactions that authors would describe in their introduction and methods sections, and the actual construction of some models. Concrete examples included misalignment of stated model goals with reported model construction and/or outcomes. For example, multiple papers stated in the introduction section that a goal of the model was to explore how HIV-related changes in host susceptibility to other pathogens may change population trends, but then nothing in the structure of the model reflected a change in host susceptibility. Similarly, authors would often expound on the importance of specific host cohorts in transmission, such as men who have sex with men or intravenous drug users, but then only explicitly include heterosexual sexual encounters in their contact process represented in the model. These overlooked errors in the pairing of model structure to known biology are worrisome in a peer-reviewed system. In addition to both authors and reviewers devoting more attention to detail, these errors suggest a need for greater emphasis on the correspondence between model structure and stated biological assumptions in training around mathematical modelling of infectious disease dynamics. In particular, when a model could be a useful tool for policymakers, scientists must take into account any relevant biology of the pathogens that may alter policy implementation, such as by focusing on specific higher risk host cohorts, and if simplifications are made in the model then those constraints and limitations should be clearly discussed in the text.

### (d) Future directions: multi-scale, multi-pathogen models

Much of the complexity of disease dynamics arises from interactions across scales of biological organization; from within hosts to the population level, and from the population to the greater ecological community, disease dynamics occur across orders of magnitude. As the field of modelling co-circulating pathogens continues to grow and evolve, the effects of scale in modelling studies cannot be minimized. New studies are needed that examine multi-scale interactions and impacts of multiple pathogens in a single model. Consider, for example, the known effect of influenza on respiratory immune defences, leading to a greater likelihood of infection by bacterial pathogens such as *Streptococcus pneumoniae* and also potentially worse clinical outcomes [54]. While a few papers in our dataset did make simple adjustments to the model to include aspects such as changes in host susceptibility due to infection, very few contained a true within-host model where the processes at play within the host had the potential to influence population-level transmission in a nuanced or mechanistic manner. Work that connects the known within-host, cellular-level impacts of pathogen–pathogen interactions [55] to population-level prevalence and disease burden could give insight into better policies about surveillance tactics and treatment schemes to mitigate the negative impacts of these pathogens. However, simply increasing model complexity by incorporating multiple scales is not sufficient to generate new insights if the biological knowledge and data available to support the model structure are not present. Key to this pursuit are appropriate data sources at multiple scales, from the individual to the population level, to verify how pathogens are interacting within hosts and tie these data to phenomena observed with co-circulating pathogen transmission at the population level. For respiratory pathogens, the COVID-19 pandemic and the accompanying ‘tripledemic’ of COVID-19, influenza and RSV has, at least temporarily, heightened testing and surveillance and expanded the use of excellent tools such as wastewater data [56–58]. We hope for a continued focus on gathering high-quality data for these and other pathogens, which will allow researchers to build robust, data-informed models that can directly inform public health efforts and policies.

### (e) Study limitations

It is important to emphasize that we restricted our review to include only models that contained a mechanistic representation of co-circulating pathogens of humans. This criterion allowed for an in-depth focus on this subset of the literature, but we recognize that there are still many insights to be found from non-mechanistic statistical and geospatial approaches, models of theoretical pathogens, and work done in animal and plant systems. Work on theoretical pathogens can explore central mechanisms that drive the patterns seen in co-circulating pathogens in human populations, such as how co-infection may (or may not) drive changes in pathogen virulence [59]. Models of theoretical pathogens can also be useful to model scenarios of an outbreak of a currently uncharacterized pathogen/ ‘Disease X’ and how it may interact with pathogens that are already endemic in a population. Experimental manipulations in non-human systems that would not be feasible or ethical with human subjects can also yield fundamental insights into the interactions and mechanisms at play in a multi-pathogen world, such as how variability in infection risk can be more strongly influenced by co-infecting pathogens than other mechanisms such as host exposure to parasites and host body condition [60]. These examples highlight the depth of investigation that is possible in other host–pathogen systems, and we want to emphasize that despite the focus of this systematic review, the power of non-human studies to inform and inspire work on human pathogens should not be discounted. A tandem review of co-circulating pathogen models that did not meet our inclusion criteria, such as non-mechanistic models and those focused on non-human pathogens, could help further inform and advance approaches across systems.

### (f) Conclusion

Co-circulating pathogen models can be a highly useful tool in the public health policy toolkit when it comes to preventing and mitigating infectious disease. From the papers in our review, we found clear examples of concrete recommendations that arise from considering co-circulating pathogens, ranging from actionable policy recommendations based on a realistic public health budget [61] to screening recommendations to meet World Health Organization targets for a particular disease [26]. We believe many more actionable insights remain to be discovered from future work on co-circulating pathogens. What level of surveillance is needed to track and forecast a pathogen of interest when it co-circulates with other pathogens that cause similar clinical symptoms, such as arboviruses? What testing and treatment regimens can minimize the population-level effects of co-circulating respiratory pathogens that are known to have within-host interactions? How might antibacterial treatment for one pathogen influence the trajectory of antibiotic resistance not just in the concerned species, but other co-circulating bacteria? The answers to these questions and others are needed to guide informed public health policy decisions, but first we must identify current knowledge gaps and the methods available to tackle them. We hope that this systematic review serves as a launching point for others to take on the most exciting and pressing challenges presented by co-circulating pathogens, and in doing so mitigate the burden of infectious diseases.

## Data Availability

A full version of the questionnaire can be found in the supplementary materials. All data were analysed in R v. 4.3.0 [32], and raw files and code used for analysis as well as a supplementary file of all tables produced from the analysis are available at https://figshare.com/s/2b268f5df8aed6acad3f. Supplementary material is avaialable online [62]
